# Fixed Drug Eruptions With Flavoured Liquid Formulations of Over-the-Counter Analgesics: A Case Report

**DOI:** 10.7759/cureus.43436

**Published:** 2023-08-13

**Authors:** Ali H Alzahrani

**Affiliations:** 1 Allergy and Immunology, King Abdulaziz University, Jeddah, SAU

**Keywords:** nsaid delayed skin reactions, inactive drug ingredient reactions, liquid paracetamol reactions, drug, type 4 hypersensitivity reaction, fixed-drug eruption, bullous fixed drug eruption

## Abstract

Type 4 hypersensitivity reactions convey a number of conditions that include fixed drug eruptions (FDEs). They share similar pathophysiologic backgrounds and sometimes presentation but can have very variable prognostications. Drugs are amongst the possible causes with acetaminophen and other NSAIDs being reported very frequently. We present a case of a patient reacting to flavoured oral ibuprofen and acetaminophen formulations, exhibiting FDEs with bullae formation. We describe our successful challenge to non-flavoured acetaminophen and ibuprofen. We briefly discuss FDEs in regard to their incidence, pathophysiology, and management.

## Introduction

Fixed drug eruptions (FDEs) are skin reactions that result from a type 4 hypersensitivity reaction potentially developing within minutes to hours or occasionally more akin to other type 4 reactions but may happen 14 days after an offending agent (for example, drugs or vaccines) [1]. In FDEs, the lesions have this tendency to reappear in the same places upon further exposure, hence the term "fixed," and at sites of previous trauma [2]. This phenomenon can be helpful in diagnosing and predicting the distribution of the reaction. It is classically described as usually being oval with a dusky colour and being well-demarcated. These lesions, however, can erupt variably and express drastically different features similar to erythema multiforme or more dramatically bullous eruptions that mimic other type 4 reactions known for worse prognoses, such as toxic epidermal necrolysis (TEN) and Steven-Johnson syndrome (SJS). The lack of mucosal involvement and the rash distribution can give diagnostic hints [3], but are not conclusive as mucosal involvement has been described [4]. Systemic symptoms can occur in a rarity, but they are more common in bullous eruptions. Diagnosis remains largely clinical though, sometimes, a biopsy is needed to differentiate, and the prognosis is generally very favourable compared to TEN/SJS. A case-control study revealed otherwise mainly in older age [3,5].

Many drugs have been described and reported, including the very commonly used NSAIDs and acetaminophen [1,6]. We could not find enough data highlighting the occurrence of over-the-counter (OTC) medications for FDE, but it has been reported for variable OTC drugs, including multivitamins [7]. We came across a case of an 11-year-old girl that demonstrated FDEs to flavoured liquid formations of NSAIDs and tylenol, but not to plain formulations of both. To our knowledge, this is the first case report demonstrating the occurrence of drug eruptions of NSAIDs/acetaminophen that successfully tolerated non-flavoured formulations upon drug challenge.

## Case presentation

An 11-year-old female has a history of previous FDE in 2019, with a suggestive biopsy finding of consuming flavoured Ibuprofen. She had bulla formations and a rash that spread over her neck and chest, which was managed with opioids, antihistamines, and topical steroids. She was scheduled for an acetaminophen challenge, but the family was reluctant.

**Figure 1:**
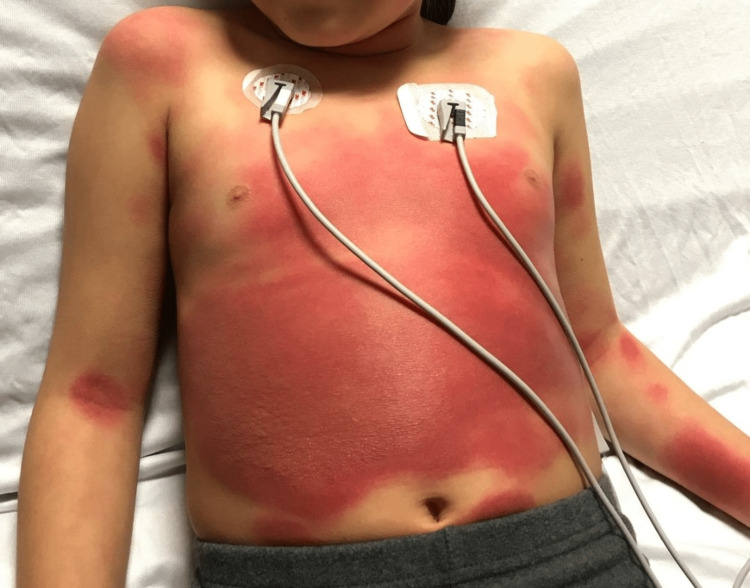
Day one of ER visit. Notable are the drug eruptions on her abdomen and upper limb. On the right side of her abdomen appears the vesicular component.

**Figure 2:**
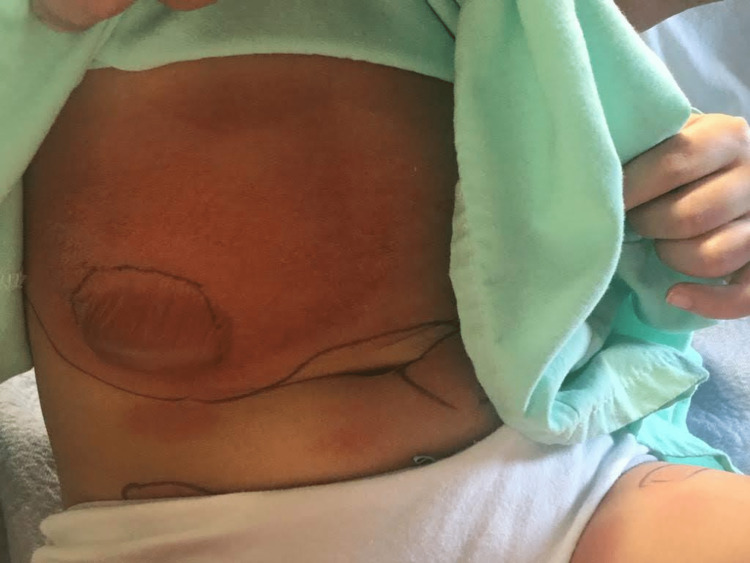
Day two following admission. The bulla is noticeable on her abdomen. It started to collect.

**Figure 3:**
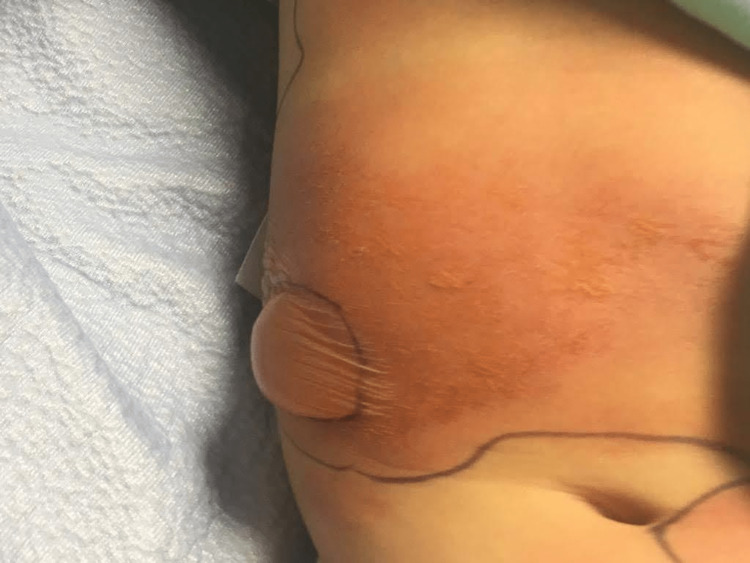
On day five, the bulla continues to collect more, and the overall improvement of her rash is noticeable.

Two years later, she had an ER visit for a viral infection. She was given non-flavoured liquid acetaminophen. She had no reaction and was discharged on non-flavoured acetaminophen with no issues at home. A month after, she went camping, and, a few days upon return, she started having a fever and rhinorrhoea. The swab for COVID-19 was negative, and the patient was given flavoured acetaminophen liquid that contained anhydrous citric acid, FD&C red no. 40 (Allura red, a dye), glycerin, high fructose corn syrup, microcrystalline cellulose, carboxymethylcellulose sodium, purified water, sodium benzoate, sorbitol solution, sucralose, and xanthan gum. Within three hours, the patient started to have a burning sensation on the sites she previously had a rash two years ago, which included her neck, chest, and abdomen where she also started to have increasing pain. The rash is composed of multiple erythematous plaques of various sizes, with vesicular/bullous components, as shown in Figure 1. She was vitally stable. She was admitted for monitoring. She had a normal ECG at that point, and her labs demonstrated a normal complete blood count, normal liver function test, and normal kidney function. Cetirizine and topical steroids were started. Within two days, she developed a bulla on the right side of her abdomen (Figure 2). The bulla was not tense but slightly painful. A dermatologist thought a biopsy was not needed given the lesions followed the expected distribution of her previous FDE. The patient continued to have no mucosal or genital involvement. The bullae continued to fill (Figure 3) before it ruptured on day six, and the patient was discharged home on topical steroids. Complete resolution of active lesions took two weeks. She is left with hyperpigmentation on her abdomen and neck.

A lymphocyte toxicity assay, an in vitro blood test, was done by a clinical pharmacist. The patient’s lymphocytes isolated from peripheral blood samples showed variable hypersensitivity results to flavoured oral formulations of acetaminophen and ibuprofen. Non-flavoured formulation showed no reaction.

We have offered them a patch test for flavoured brands she reacted with, in addition non-flavoured ones. We thought this was the safest plan given that her reactions were more generalized and necessitated admission. Unfortunately, due to the acuity of their schedule, they declined. We discussed with them the safety and concerns of doing a challenge. Given that the lymphocyte toxicity assay showed a complete lack of reaction to non-flavoured formulations of both medications, we decided to challenge both medications. We started with the non-flavoured liquid ibuprofen challenge, and we aimed for a total dose of 100 mg. We decided to start with one-tenth of a dose, starting at 10 mg, and watched clinically for three hours. Vitals were taken at the start. She was frequently inspected for any skin rashes. She had no issue and was given the remaining dose and kept for three more hours with frequent inspection. In the end, she reported no reaction to the challenge and was completely normal. We have called her twice over two weeks with no issues. She came to the clinic again for a one-step non-flavoured liquid acetaminophen challenge that she had no reaction to. She reported no issues upon virtual follow-ups. We advised her to take non-flavoured formulations of the OCT medications as of now and to reconsider patch testing to flavoured formulations when that is convenient. Our aim is to try an elicit possible ingredient, other than the active medications causing the reaction.

## Discussion

FDEs remain a rare medical entity that composed around 0.003% of total skin reactions in a US-based study that included 2.7 million individuals [8]. It is, however, reported more frequently in smaller series studies. Two studies showed 14.1% over a series of 90 patients in a study conducted in Tunisia [9] and 22% over 50 patients in a study conducted in India [10]. While drugs are the most common cause, other culprits such as vaccines and food have been prescribed [1]. They also tend to occur at sites of previous trauma or injury, suggesting a recall phenomenon [2]. They usually have a good prognosis, but not always, and can lead to local complications, as well such as eyelid necrosis [11]. 

The pathophysiology of FDEs is majorly driven by intraepidermal CD8+ cells found in active and inactive lesions [12]. These cells, upon reactivation with a causative agent, trigger interferon-gamma production in addition to cytotoxic granules and granzyme B contributing to the development of skin lesions.

Histopathology for FDEs differs depending on the stage and occasionally can be inconclusive. If the biopsy was taken at an active lesion, it shows basal keratinocyte degradation and lymphocytic infiltration in the dermis. A biopsy of resting lesions shows numerous CD8 lymphocytes in the dermo-epidermal junction that moves upward while maintaining a normal-looking dermis two to three hours upon being triggered, and then after 48 hours, the active features appear. There are other atypical presentations, including leukocytoclastic vasculitis [13]. It has been shown in studies that primed CD8 cells can survive in the absence of a trigger for more than four years, further explaining the repeated involvement of the same skin sites upon reactivation by a trigger [14].

While acetaminophen and NSAIDs are thought to be one of the most common triggers for the variable forms of FDEs [1,6], other culprits, including antibiotics and anti-epileptics, are also reported with variable frequency. Dyes and colouring agents were implicated in many reactions, including both immediate and delayed [15,16], but no specific case reports with successful challenges. There have been variable reports about the reactions, ranging from the typical presentation to other unique but individual presentations, such as the aforementioned eyelid necrosis [11]. 

Diagnosing FDEs remains largely clinical owing to the typical presentation and the usually fixed re-involvement of the previously affected areas. A biopsy is helpful, but is not always indicated, especially in the complete lack of worrisome or atypical features or when there are high suspicions of TEN or SJS.

A lymphocyte toxicity assay is a novel diagnostic test that has been adapted to variable extents clinically to help identify a causative agent for delayed drug reactions [17]. The idea of this in vitro test is to see how sensitive lymphocytes of an individual are unable to detoxify the susceptible drug applied in vitro. It has garnered some evidence, but for the most part, it has remained largely a supportive test, as in studies that demonstrated variable sensitivity and specificity [18]. It remains not largely available and uncommon [17]. Multiple case reports have demonstrated its use [19,20]. It does not substitute clinical history, patch testing, or oral challenge if feasible to diagnose type 4 reactions. In our case, it was done beforehand by a clinical pharmacist when they were consulted. While the results were consistent with our clinical history, it was the fact that she tolerated non-flavoured acetaminophen given to her in an ER visit, which prompted us to offer the oral challenge.

Provocation challenges are advisable if the culprit drug cannot be identified clinically, in cases where more are suspected. It is, however, contraindicated in the presence of severe systemic symptoms or if SJS and TEN were highly suspected and not ruled out. However, no way has been standardized, and the practice depends largely on the physician's experience. A recommended way is to start with a 1/10th and then increase the dose to a full dose every 12-24 hours. Another approach is to give half a dose and then increase it to a full dose if no reaction occurs. In our approach, we managed with a 1/10th of a dose, with the remaining dose given in three hours while watching her for three more hours. Another approach that we considered was to space the challenge over a two-day course, but the patient refused given the time constraint. With our non-flavoured acetaminophen, we administered it in one dose and followed her for half a clinic day (four hours) as she has been given that before with no reaction and our challenge was to affirm that.

Patch testing remains largely non-standardized for the most part and follows different protocols. The reactions would be localized. We wanted to mix the flavoured liquid medications with petrolatum and have it occluded on her skin; if erythema developed within 24 hours and lasted more than six hours, the test is positive. This could have helped prove the reactivity and usability of the patch test to her known two medications; after which point, we could have added the inactive ingredients that are available with other OTC medications and see if she will have positives as well. This will help us avoid further possible reactions and give us clues about the possible inactive ingredient/component leading to her reaction. Of note, the test is limited by a high degree of false negatives (reaching 40%), especially when applied to non-involved areas [21], but remains a very useful test if used in the right clinical setting.

Fixed bullous drug eruption treatment follows the principles of type 4 reactions, where the first step is to stop all agents implied and then to treat symptomatically. Care should be given to differentiate between the different type 4 reactions. Itching can be managed with H1 anti-histamines and topical variable potency steroids. In case of oral involvement, local analgesics, such as lidocaine, can be utilized. In case of pain, appropriate analgesia should be given. Systemic immunosuppressants, such as cyclosporine, are indicated sometimes in the case of generalized diseases [17].

## Conclusions

FDEs are one of the type 4 hypersensitivity reactions. It can present variably and be mistaken for other drug reactions or primary skin conditions. It usually has a good clinical outcome. NSAIDs and acetaminophen remain among the most common causes of FDEs, but as in our case, we wonder if those reactions are sometimes caused by components of different formulations of these medications, other than the active ingredients. Considering this, appropriate and delicate clinical assessment using patch testing and oral challenge, if possible, would help keep these otherwise safe, affordable, and commonly needed medications as an available option.

## References

[1] McClatchy J, Yap T, Nirenberg A, Scardamaglia L (2022). Fixed drug eruptions - the common and novel culprits since 2000. J Dtsch Dermatol Ges.

[2] Mizukawa Y, Shiohara T (2002). Trauma-localized fixed drug eruption: involvement of burn scars, insect bites and venipuncture sites. Dermatology.

[3] Cho YT, Lin JW, Chen YC, Chang CY, Hsiao CH, Chung WH, Chu CY (2014). Generalized bullous fixed drug eruption is distinct from Stevens-Johnson syndrome/toxic epidermal necrolysis by immunohistopathological features. J Am Acad Dermatol.

[4] Ferreira C, Corrales T, Guilherme A (2020). Fixed drug eruption on the tongue due to naproxen. J Investig Allergol Clin Immunol.

[5] Lipowicz S, Sekula P, Ingen-Housz-Oro S (2013). Prognosis of generalized bullous fixed drug eruption: comparison with Stevens-Johnson syndrome and toxic epidermal necrolysis. Br J Dermatol.

[6] Brahimi N, Routier E, Raison-Peyron N (2010). A three-year-analysis of fixed drug eruptions in hospital settings in France. Eur J Dermatol.

[7] Jha N (2020). Bullous fixed drug eruption related to multivitamins. Dermatol Online J.

[8] Wong A, Seger DL, Lai KH (2019). Drug hypersensitivity reactions documented in electronic health records within a large health system. J Allergy Clin Immunol Pract.

[9] Khaled A, Kharfi M, Ben Hamida (2012). Cutaneous adverse drug reactions in children. A series of 90 cases. Tunis Med.

[10] Sharma VK, Dhar S (1995). Clinical pattern of cutaneous drug eruption among children and adolescents in north India. Pediatr Dermatol.

[11] Kimmatkaar P, Das S, Gandhi A, Taneja V (2018). Paracetamol-induced fixed drug eruption presenting as eyelid skin necrosis. Indian J Ophthalmol.

[12] Mizukawa Y, Shiohara T (2009). Fixed drug eruption: a prototypic disorder mediated by effector memory T cells. Curr Allergy Asthma Rep.

[13] Harris A, Burge SM (1995). Vasculitis in a fixed drug eruption due to paracetamol. Br J Dermatol.

[14] Mizukawa Y, Yamazaki Y, Shiohara T (2008). In vivo dynamics of intraepidermal CD8+ T cells and CD4+ T cells during the evolution of fixed drug eruption. Br J Dermatol.

[15] Tattersall I, Reddy BY (2016). Fixed drug eruption due to achiote dye. Case Rep Dermatol.

[16] Caballero ML, Quirce S (2020). Delayed hypersensitivity reactions caused by drug excipients: a literature review. J Investig Allergol Clin Immunol.

[17] Patel S, John AM, Handler MZ, Schwartz RA (2020). Fixed drug eruptions: an update, emphasizing the potentially lethal generalized bullous fixed drug eruption. Am J Clin Dermatol.

[18] Elzagallaai AA, Jahedmotlagh Z, Del Pozzo-Magaña BR (2010). Predictive value of the lymphocyte toxicity assay in the diagnosis of drug hypersensitivity syndrome. Mol Diagn Ther.

[19] Kim MH, Shim EJ, Jung JW, Sohn SW, Kang HR (2012). A case of allopurinol-induced fixed drug eruption confirmed with a lymphocyte transformation test. Allergy Asthma Immunol Res.

[20] Demir S, Cetin EA, Unal D (2018). Generalized fixed drug eruption induced by fluconazole without cross-reactivity to itraconazole: lymphocyte transformation test confirms the diagnosis. Drug Saf Case Rep.

[21] Phillips EJ, Bigliardi P, Bircher AJ (2019). Controversies in drug allergy: testing for delayed reactions. J Allergy Clin Immunol.

